# UBE2S interacting with TRIM28 in the nucleus accelerates cell cycle by ubiquitination of p27 to promote hepatocellular carcinoma development

**DOI:** 10.1038/s41392-020-00432-z

**Published:** 2021-02-16

**Authors:** Ren-Yu Zhang, Ze-Kun Liu, Ding Wei, Yu-Le Yong, Peng Lin, Hao Li, Man Liu, Nai-Shan Zheng, Ke Liu, Cai-Xia Hu, Xiao-Zhen Yang, Zhi-Nan Chen, Huijie Bian

**Affiliations:** 1 National Translational Science Center for Molecular Medicine, Department of Cell Biology, Fourth Military Medical University, Xi’an, 710032 China; 2 School of Life Sciences, Central China Normal University, Wuhan, 430079 China; 3 Oncology and Hepatobiliary Minimally Invasive Interventional Center, Beijing Youan Hospital, Capital Medical University, Beijing, 100069 China

**Keywords:** Tumour biomarkers, Oncogenes

## Abstract

Genomic sequencing analysis of tumors provides potential molecular therapeutic targets for precision medicine. However, identifying a key driver gene or mutation that can be used for hepatocellular carcinoma (HCC) treatment remains difficult. Here, we performed whole-exome sequencing on genomic DNA obtained from six pairs of HCC and adjacent tissues and identified two novel somatic mutations of UBE2S (p. Gly57Ala and p. Lys63Asn). Predictions of the functional effects of the mutations showed that two amino-acid substitutions were potentially deleterious. Further, we observed that wild-type UBE2S, especially in the nucleus, was significantly higher in HCC tissues than that in adjacent tissues and closely related to the clinicopathological features of patients with HCC. Functional assays revealed that overexpression of UBE2S promoted the proliferation, invasion, metastasis, and G1/S phase transition of HCC cells in vitro, and promoted the tumor growth significantly in vivo. Mechanistically, UBE2S interacted with TRIM28 in the nucleus, both together enhanced the ubiquitination of p27 to facilitate its degradation and cell cycle progression. Most importantly, the small-molecule cephalomannine was found by a luciferase-based sensitive high-throughput screen (HTS) to inhibit UBE2S expression and significantly attenuate HCC progression in vitro and in vivo, which may represent a promising strategy for HCC therapy.

## Introduction

Cancer is a group of abnormal genomic diseases characterized by acquired genetic changes such as point mutations, amplifications, deletions, translocations, etc.^1^ The liver is the central organ involved in metabolism and detoxification, and is often exposed to endogenous and exogenous mutagens.^2^ Recent studies have explored hepatocellular carcinoma (HCC) genomic alterations and have identified a series of high-frequency driver genes/mutations or pathways, including TP53/cell cycle pathway, Wnt/β-catenin pathway, and telomere maintenance.^2,3^ These mutational signatures are of great significance for people to understand the association between genetic/epigenetic changes and tumor etiology, and elucidate the mechanisms of tumorigenesis. Hepatocarcinogenesis is partly due to the disorder of cell cycle, thus promoting the proliferation of HCC cells.^4^ Galina Khemlina et al.^5^ summarized key genes deletions (CDKN2A) or amplifications (CCND1, FGF3, FGF4, and FGF19) in cell cycle pathways during liver carcinogenesis, and these aberrant signals may provide a potential source of molecular targets for new therapies. However, identifying a key driver gene or mutation that can both participate in the cell cycle progression and be effectively applied in the treatment of HCC remains difficult.

The ubiquitin-proteasome pathway consists of E1 ubiquitin-activating enzymes (E1s), E2 ubiquitin-conjugating enzymes (E2s), and E3 ubiquitin ligases (E3s), and is associated with tumorigenesis.^6^ The human genome encodes two E1s, at least 38 E2s and >600 E3s.^7–9^ In eukaryotic cells, target proteins are modified with monoubiquitin or polyubiquitin chains linked via the N-terminus of any of seven lysine (K) residues of ubiquitin (K6, K11, K27, K29, K33, K48, and K63).^10^ These ubiquitin chain configurations determine an array of protein functions, including localization, proteasome degradation, and signal transduction.^11,12^

UBE2S is a member of the E2 family of ubiquitin-conjugating enzymes, and has been reported to cooperate with anaphase-promoting complex/cyclosome (APC/C) to elongate K11-linkages and polyubiquitin chains on substrates for 26 S proteasome-mediated degradation.^13,14^ In addition, UBE2S and RNF8 E3 ligases modify damaged chromatin via K11-linkages to regulate DNA damage-induced transcription silencing.^15^ Interestingly, UBE2S is also responsible for Lys11-linkage ubiquitin modifications on β-catenin, which antagonizes complex/β-TrCP cascade-orchestrated β-catenin degradation, activating Wnt/β-catenin signaling.^16^ Previous studies have reported that UBE2S is up regulated in several human cancers and negatively correlates with the clinical outcome of cancer patients.^17–20^ Overexpression of UBE2S promotes tumor growth and metastasis by targeting the von Hippel-Lindau tumor suppressor (pVHL) and p53 for degradation.^20,21^ Reduction of UBE2S expression inhibits the invasion of cervical cancer by regulating epithelial-mesenchymal transition (EMT) signaling.^22^ However, the key mechanisms of UBE2S in promoting HCC growth are poorly understood.

Tripartite motif-containing 28 (TRIM28), also known as transcriptional intermediary factor 1 β (TIF1-β) or Krüppel-associated box (KRAB)-associated protein 1 (KAP1), is a member of the tripartite motif-containing (TRIM) protein family.^23^ TRIM28 contains N-terminal TRIM and C-terminal PHD-bromo domains, and the tripartite motif is composed of a RING domain, two B-box domains, and a coiled-coil region.^24,25^ Previous studies have shown that TRIM28 not only functions as an E3 ubiquitin ligase targeting p53 and AMPK for degradation through proteasome-dependent pathways, but acts as a transcriptional regulator interacting with SETDB1 and HDAC1 to facilitate transcriptional repression.^26,27^ In addition, TRIM28 is highly expressed in various tumors including glioma, non-small cell lung cancer, breast cancer, gastric cancer, and HCC.^28–30^ TRIM28 promotes tumor cell proliferation and metastasis and serves as an important prognostic indicator of cancer.^31^ However, the mechanism through which TRIM28 promotes the growth of HCC cells remains poorly characterized.

In this study, we demonstrate that UBE2S is highly expressed in HCC, particularly in the nucleus, and is closely related to the clinical prognosis of HCC patients. Moreover, UBE2S can enter the nucleus through its nuclear localization signal (NLS), where it interacts with TRIM28, and enhances the ubiquitination of p27, thereby promoting HCC progression. Finally, we screened a small molecule library and identified cephalomannine as a compound that can significantly reduce UBE2S expression and inhibit the growth of HCC cells. These data highlight new diagnostic and therapeutic strategies for the clinical treatment of HCC patients.

## Results

### Identification of candidate somatic mutations through WES

We performed whole-exome sequencing (WES) of genomic DNA obtained from six pairs of punctured HCC and adjacent non-tumor tissues. The pipeline for variant analysis of cancer WES data was previously described.^32^ Single nucleotide variations, insertions, and deletions (indels) were analyzed using the three-caller pipeline to identify somatic mutations through the comparison of variants identified in tumor exome data sets against germline variants and dbSNP in paired adjacent samples.^32^ A somatic mutant UBE2S with two different mutation sites (p.Gly57Ala and p.Lys63Asn) were identified in a single patient. Both alterations were validated in the original samples using IGV visualization and PCR-based Sanger sequencing of tumor and paired adjacent tissue DNA from the same patient (Supplementary Fig. 1a). Both mutations were heterozygous and led to amino-acid substitutions (Supplementary Fig. 1a). By aligning the sequences of homologous UBE2S among different species, we found that the Gly57 and Lys63 residues were highly conserved throughout evolution (Supplementary Fig. 1b). Predictions of the functional effects of the mutations showed that two amino-acid substitutions were potentially deleterious (Supplementary Fig. 1c). The somatic mutations of UBE2S in HCC reported in the COSMIC database, ICGC database and in this study were summarized in Supplementary Fig. 1d.

### UBE2S is upregulated in human HCC tissue and associated with poor prognosis and hepatocarcinogenesis

**Fig. 1:**
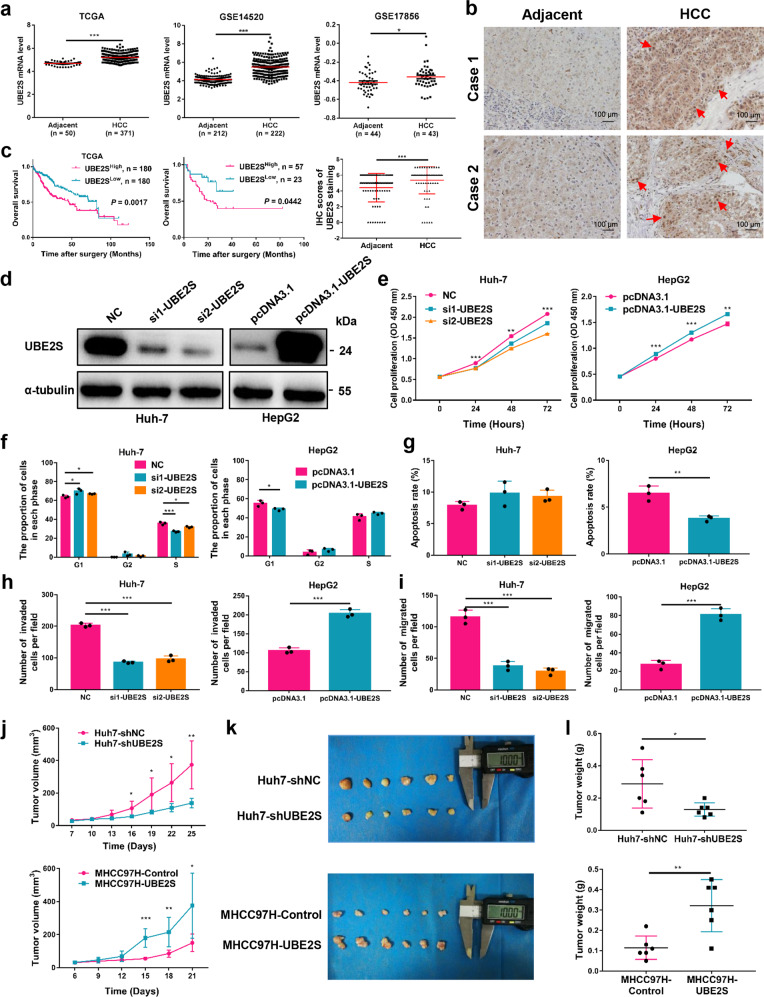
UBE2S is an indicator of poor prognosis and promotes HCC progression. **a** Expression of UBE2S in HCC and adjacent tissues in TCGA, GSE14520 and GSE17856 data sets. **b** Immunohistochemical detection of UBE2S expression in human HCC and adjacent tissue (*n* = 80 pairs). Red arrows indicate UBE2S in nucleus. **c** OS rates of HCC patients with high or low UBE2S expression were evaluated by Kaplan–Meier analysis using TCGA data sets and 80 cases human HCC tissues. **d** Western blot analysis of UBE2S in HCC cells transfected with siRNA or overexpression vectors. **e–i** Effects of UBE2S on cell proliferation, cell cycle progression, apoptosis, invasion, and migration detected by CCK-8, flow cytometry, and transwell assays, respectively. **j** Effects of UBE2S on tumor growth in xenograft nude mouse models. Stably transfected cells were subcutaneously inoculated on the back of nude mice (5 × 10^6^ Huh-7 or 3 × 10^6^ MHCC-97H cells/mice, *n* = 6). Tumor volumes were measured after 7 days (Huh-7) or 6 days (MHCC-97H) of inoculation and every three days thereafter. **k** Tumor tissues in each group. **l** Tumor weights were measured on days 25 (Huh-7) or 21 (MHCC-97H). Two-tailed Student’s *t* tests were used to test the significance of differences between two groups; data are represented as mean ± SEM (**a**–**b**, **e**–**j**, **l**). Kaplan–Meier curves of overall survival of HCC patients were determined by the log-rank test (**c**). *P < 0.05, **P < 0.01, ***P < 0.001

To explore the role of UBE2S in HCC, we analyzed its mRNA expression in HCC and adjacent non-tumor tissues using the TCGA database, GSE14520 and GSE17856 data sets. The mRNA levels of UBE2S in HCC tissue were significantly higher than adjacent tissues (Fig. 1a). Furthermore, we analyzed UBE2S expression in a cohort of 80 pairs of HCC and adjacent tissues with immunohistochemistry, and observed higher levels of UBE2S in HCC compared with adjacent tissues (Fig. 1b). We further analyzed the relationship between UBE2S expression and the clinical prognosis of HCC patients based on TCGA database and immunohistochemistry analysis. The results showed that HCC patients with high UBE2S mRNA and protein levels had shorter overall survival (OS) rates compared with those with low UBE2S expression (Fig. 1c). UBE2S also positively correlated with tumor number, tumor size, and tumor-node metastasis stage (Table 1). These results suggest that UBE2S has a key role in HCC development.

**Table 1:** Correlation of UBE2S expression and clinicopathologic features in patients with hepatocellular carcinoma

Clinicopathological variables	UBE2S expression		*P* value	Nuclear UBE2S expression		*P* value
	High (*n* = 57)	Low (*n* = 23)		Positive (*n* = 41)	Negative (*n* = 39)	
*Gender*						
Male	47	22	0.233	33	36	0.125
Female	10	1		8	3	
*Age*						
<50 years	22	10	0.687	14	18	0.273
≥*50 years*	35	13		27	21	
HBsAg						
Positive	49	21	0.779	35	35	0.800
Negative	8	2		6	4	
*Serum AFP*						
<20 (ng/ml)	17	9	0.421	13	13	0.877
≥20 (ng/ml)	40	14		28	26	
*Tumor number*						
Single	37	20	**0.049**	25	32	**0.037**
Multiple	20	3		16	7	
*Tumor size*						
<5 cm	17	15	**0.003**	10	22	**0.003**
≥5 cm	40	8		31	17	
*TNM stage*						
1	32	19	**0.026**	23	28	0.144
2–4	25	4		18	11	
*Tumor differentiation*						
I–II	22	10	0.687	16	16	0.855
III–IV	35	13		25	23	

*HbsAg* hepatitis B virus surface antigen, *AFP* alpha-fetoprotein, *TNM* tumor-node metastasis. Values in bold indicate statistically significant differences

To evaluate the role of UBE2S in HCC, we assessed its expression in different HCC and normal hepatic cell lines (Supplementary Fig. 2a). Knockdown and overexpression performances were conducted in Huh-7 and HepG2 cell lines, respectively (Fig. 1d). Moreover, we established two stable cell lines, MHCC-97H-shUBE2S and MHCC-97H-UBE2S through lentivirus infection (Supplementary Fig. 2b). CCK-8 assays showed that UBE2S silencing inhibited cell proliferation, whereas UBE2S overexpression enhanced proliferation (Fig. 1e; Supplementary Fig. 2c). Cell cycle analysis indicated that G1/S phase transition was reduced in Huh-7 cells by UBE2S silencing, and G1 arrest was reduced in HepG2 cells following UBE2S overexpression (Fig. 1f; Supplementary Fig. 3a). Apoptosis assays showed that the percentage of apoptotic cells significantly decreased in HepG2 cells overexpressing UBE2S (Fig. 1g; Supplementary Fig. 3b). In addition, transwell assays demonstrated that UBE2S knockdown suppressed the invasion and migration of HCC cells, whereas UBE2S overexpression promoted invasion and migration (Fig. 1h, i; Supplementary Fig. 2d; Supplementary Fig. 3c).

To further explore the role of UBE2S in vivo, xenograft nude mouse models were constructed by the subcutaneous inoculation of Huh-7-shUBE2S and MHCC-97H-UBE2S cells. As shown in Fig. 1j–l, UBE2S silencing significantly reduced tumor volume and weight, whereas UBE2S overexpression promoted tumorigenesis in xenograft mice. The different expression levels of UBE2S and Ki-67 in tumor tissues were examined by immunohistochemical staining (Supplementary Fig. 4). Taken together, these data suggest that UBE2S significantly promotes hepatocarcinogenesis in vitro and in vivo.

### NLS traffic UBE2S to the nucleus

**Fig. 2:**
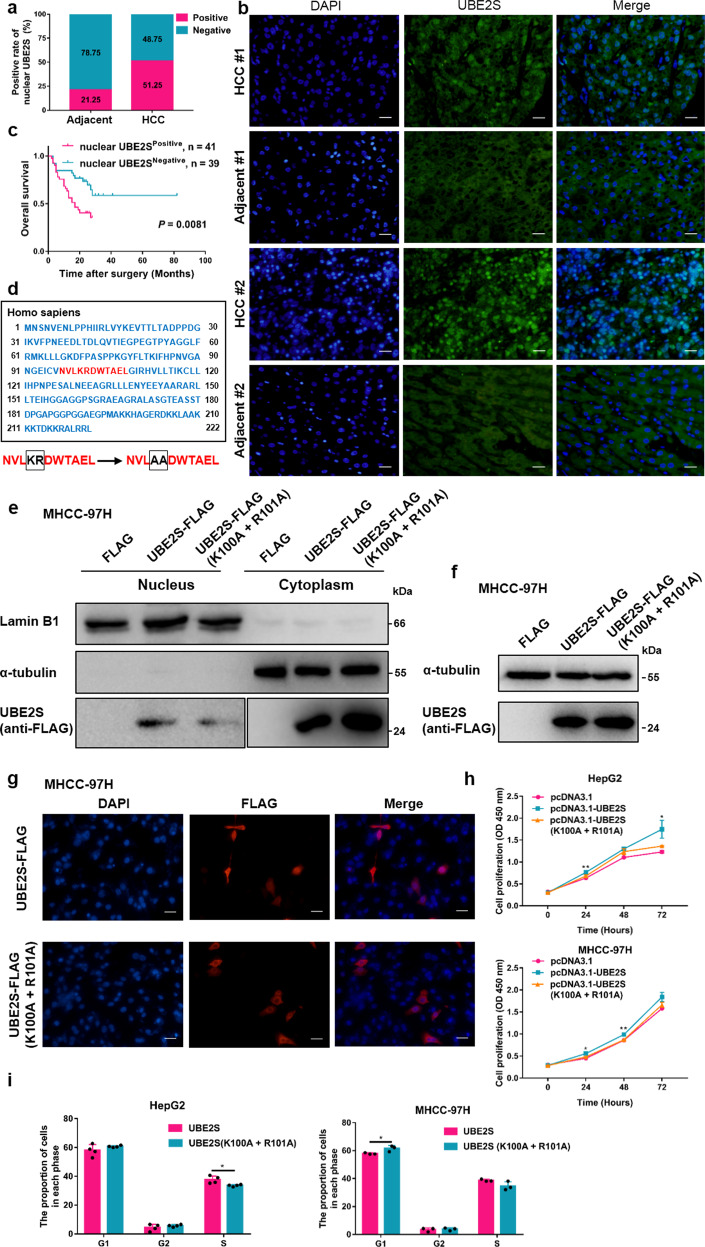
UBE2S enters the nucleus through nuclear localization signals and promotes HCC proliferation. **a** The positive rate of nuclear UBE2S in human HCC and adjacent tissues evaluated by immunohistochemistry. (*n* = 80 pairs). **b** Immunofluorescence detection of UBE2S expression in human HCC and adjacent tissues. Scale bars: 100 μm. **c** OS rates of HCC patients with positive or negative nuclear UBE2S content were evaluated by Kaplan–Meier analysis (*n* = 80 cases). **d** Bioinformatics analysis of the NLS in UBE2S and a strategy for its mutational analysis. **e**–**f** UBE2S-FLAG expression in the nuclear fractions of cell lysates and total lysates in MHCC-97H cells transiently transfected with UBE2S-FLAG NLS mutation plasmids assessed by western blotting. **g** Distribution of UBE2S-FLAG in MHCC-97H cells following mutation of the NLS detected by immunofluorescence analysis. **h**–**i** Effects of NLS mutations in UBE2S on cell proliferation and cell cycle progression determined by CCK-8 and flow cytometry assays. Two-tailed Student’s *t* tests were used to test the significance of differences between two groups; data are represented as mean ± SEM (**h**–**i**). Kaplan–Meier curves of overall survival of HCC patients were determined by the log-rank test (**c**). *P < 0.05, **P < 0.01

Subcellular distribution of UBE2S was observed in nucleus and cytoplasm (Fig. 1b). Particularly, nuclear UBE2S content was observed in 41 of 80 (51.25%) primary HCC tissues, compared with 17 of 80 (21.25%) adjacent non-tumor tissues (*P* < 0.01) (Fig. 2a). The nuclear colocalization of UBE2S in tissue comparing with the surrounding liver was further confirmed by immunofluorescence assay (Fig. 2b). As shown in Fig. 2c, HCC patients with positive nuclear UBE2S content had shorter OS rates than those with negative expression (*P* = 0.0081). Furthermore, nuclear UBE2S also positively correlated with tumor number (*P* = 0.037) and tumor size (*P* = 0.003) (Table 1).

As nuclear UBE2S content significantly increased in HCC tissues relative to adjacent tissues, we hypothesized that nuclear UBE2S has a key role during HCC progression. The amino-acid sequence of UBE2S was analyzed with cNLS Mapper (http://nls-mapper.iab.keio.ac.jp/cgi-bin/NLS_Mapper_form.cgi), which predicted an NLS sequence at position 97–107 (Fig. 2d). Two key amino-acid residues were mutated (K100A and R101A) to investigate the effect of the NLS on the subcellular localization of UBE2S (Fig. 2d). Western blot analysis showed that nuclear mutant UBE2S content in MHCC-97H cells significantly decreased (Fig. 2e), whereas total UBE2S content remained unchanged (Fig. 2f). These results were further confirmed in HEK293T cells (Supplementary Fig. 5). In addition, immunofluorescence assays confirmed that nuclear UBE2S content significantly decreased in UBE2S-mutant transfected MHCC-97H cells (Fig. 2g). Furthermore, CCK-8 and cell cycle analysis showed that mutation of the NLS in HCC cells led to reduced cell proliferation and reduced G1/S phase transition or enhanced G1 arrest (Fig. 2h, i). In summary, these data demonstrate that UBE2S has a key role in the nucleus where it promotes HCC cell growth through its regulation of cell cycle progression.

### UBE2S interacts with TRIM28 in the nucleus

**Fig. 3:**
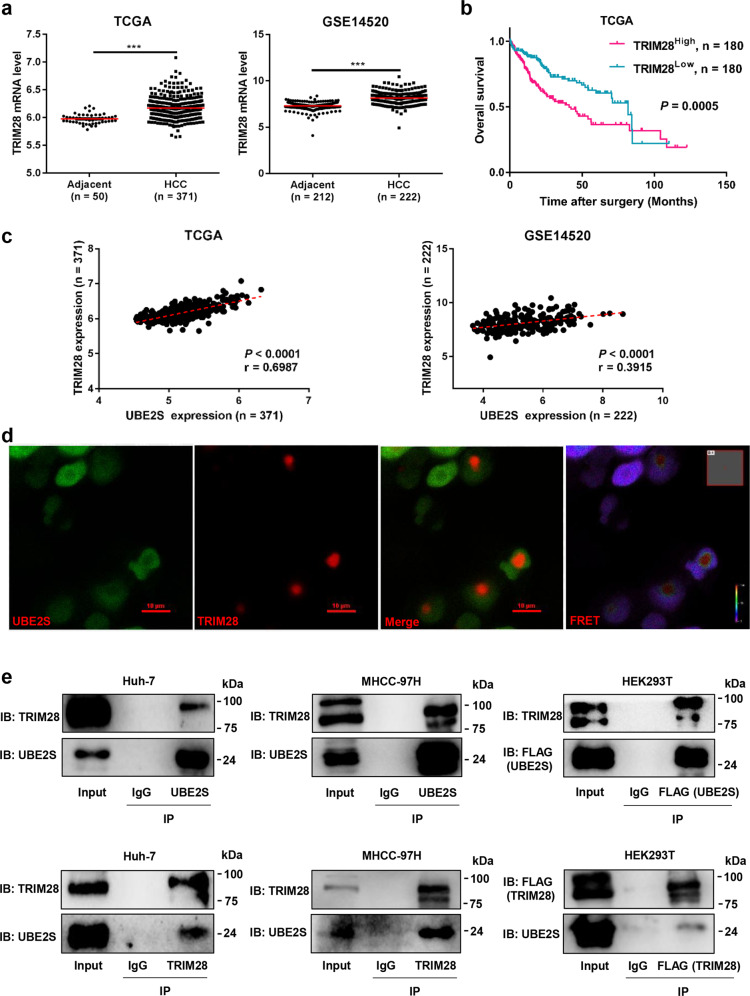
UBE2S interacts with TRIM28 in the nucleus. **a** Expression of TRIM28 in HCC and adjacent tissues in TCGA and GSE14520 data sets. **b** OS rates of HCC patients with high or low TRIM28 expression evaluated by Kaplan–Meier analysis using TCGA data sets. **c** Correlation of UBE2S and TRIM28 expression in TCGA and GSE14520 data sets. **d** Interaction between UBE2S and TRIM28 in HEK293T cells transiently transfected with UBE2S-EGFP and TRIM28-DsRed2 plasmids detected by FRET. **e** Endogenous or exogenous interaction of UBE2S and TRIM28 detected by co-immunoprecipitation and western blot analysis. Two-tailed Student’s *t* tests were used to test the significance of differences between two groups (**a**). Kaplan–Meier curves of overall survival of HCC patients were determined by the log-rank test (**b**). Pearson’s correlation test was used to assess the correlation between UBE2S mRNA expression and TRIM28 mRNA expression (**c**). ***P < 0.001

To explore the molecular mechanisms by which nuclear UBE2S promotes HCC, nuclear, and cytoplasmic fractions of Huh-7 cells were separated (Supplementary Fig. 6). Co-immunoprecipitation combined with mass spectrometry analysis of UBE2S-interacting complexes in the nuclear extracts was performed and a large number of novel binding partners including TRIM28 were identified (Supplementary Table 1). Furthermore, we found that the mRNA levels of TRIM28 in HCC tissue significantly increased compared with that of adjacent tissues in the TCGA database and GSE14520 data sets (Fig. 3a). Moreover, HCC patients with high TRIM28 expression showed lower OS rates than those in the low TRIM28 expression group (Fig. 3b). More importantly, the mRNA expression of TRIM28 in the TCGA database and GSE14520 data sets positively correlated with UBE2S (Fig. 3c). Next, we performed fluorescence resonance energy transfer (FRET) assays and found that UBE2S interacted with TRIM28 in the nucleus, but not the cytoplasm (Fig. 3d). Moreover, we confirmed the interaction between endogenous or exogenous UBE2S and TRIM28 in a range of cell lines by co-immunoprecipitation assays (Fig. 3e). In particular, the interaction between endogenous UBE2S and TRIM28 in nuclear extract of Huh-7 and MHCC-97H cells was also confirmed by co-immunoprecipitation assays (Supplementary Fig. 7). Taken together, these data indicate that UBE2S interacts with TRIM28 in the nucleus.

### The UBE2S and TRIM28 interaction regulates cell cycle progression by enhancing p27 ubiquitination

**Fig. 4:**
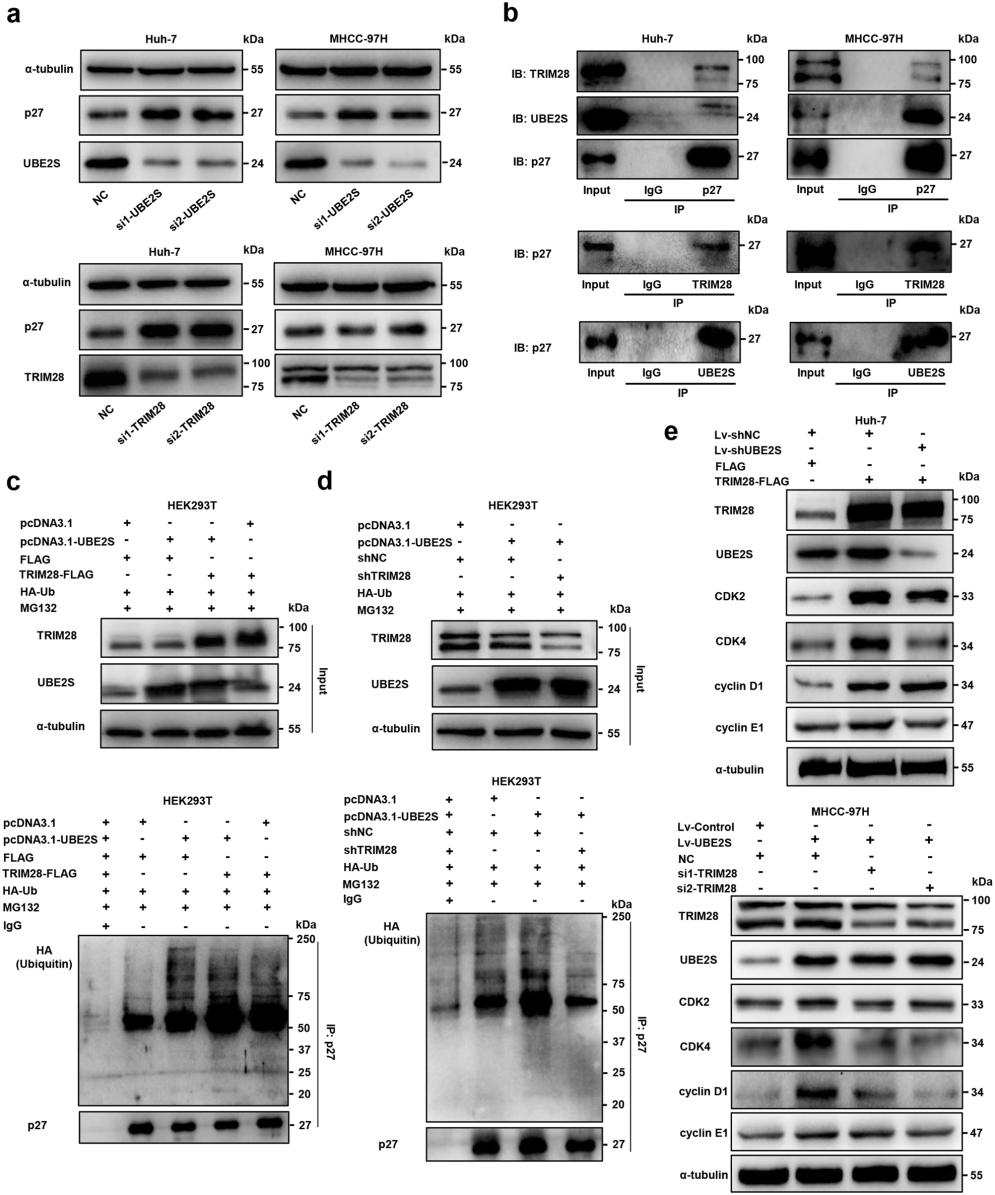
UBE2S and TRIM28 enhance the ubiquitination of p27 and regulate cell cycle progression. **a** Expression of p27 assessed by western blot analysis in HCC cells with UBE2S or TRIM28 silencing. **b** Endogenous interaction of UBE2S, TRIM28 and p27 in HCC cells. **c–d** Ubiquitination of p27 in HEK293T cells with UBE2S and TRIM28 overexpression or knockdown in the presence of MG132 (10 μM, 12 h) detected by immunoprecipitation and western blot analysis. **e** Differential expressions of cell cycle markers, including CDK2, CDK4, cyclin D1, and cyclin E1, were evaluated in HCC cells in which UBE2S and TRIM28 were elevated or silenced

To further understand the downstream pathways of UBE2S and TRIM28 in HCC cells, we focused on the mechanism of these two molecules in cell cycle regulation. Interestingly, we found that UBE2S or TRIM28 silencing significantly increased the level of p27, a cyclin-dependent kinase inhibitor, in Huh-7 and MHCC-97H cells (Fig. 4a). Moreover, Co-immunoprecipitation assays showed that both UBE2S and TRIM28 could interact with p27 in HCC cells (Fig. 4b; Supplementary Fig. 7). In particular, UBE2S silencing in HEK293T cells significantly reduced p27 ubiquitination (Supplementary Fig. 8a), whereas the overexpression of UBE2S and TRIM28 in HEK293T cells remarkably enhanced p27 ubiquitination in the presence of MG132, a commonly used proteasome inhibitor, for 12 h (Fig. 4c; Supplementary Fig. 8b). Further, the ubiquitination of p27 was increased when UBE2S was higher in HEK293T cells, and decreased when TRIM28 was deleted (Fig. 4d; Supplementary Fig. 8c). In addition, the expressions of G1/S phase transition-associated proteins, including CDK2, CDK4, cyclin D1, and cyclin E1 were significantly higher after the overexpression of TRIM28, which were downregulated following UBE2S silencing in Huh-7 cells, except for cyclin D1 (Fig. 4e). Similarly, the expressions of these cell cycle-associated proteins were increased when UBE2S was higher in MHCC-97H cells, which were decreased by TRIM28 knockdown (Fig. 4e). Most importantly, we performed p27 rescue experiments and found that G1/S phase transition was increased in Huh-7 cells through TRIM28 overexpression or in MHCC-97H cells following UBE2S overexpression, which was reduced by p27 overexpression (Supplementary Fig. 9a, b). Taken together, these data highlight that UBE2S and TRIM28 enhance the ubiquitination of p27 and promote its degradation, thus regulating cell cycle progression in HCC cells.

### Cephalomannine inhibits UBE2S expression and HCC growth

**Fig. 5:**
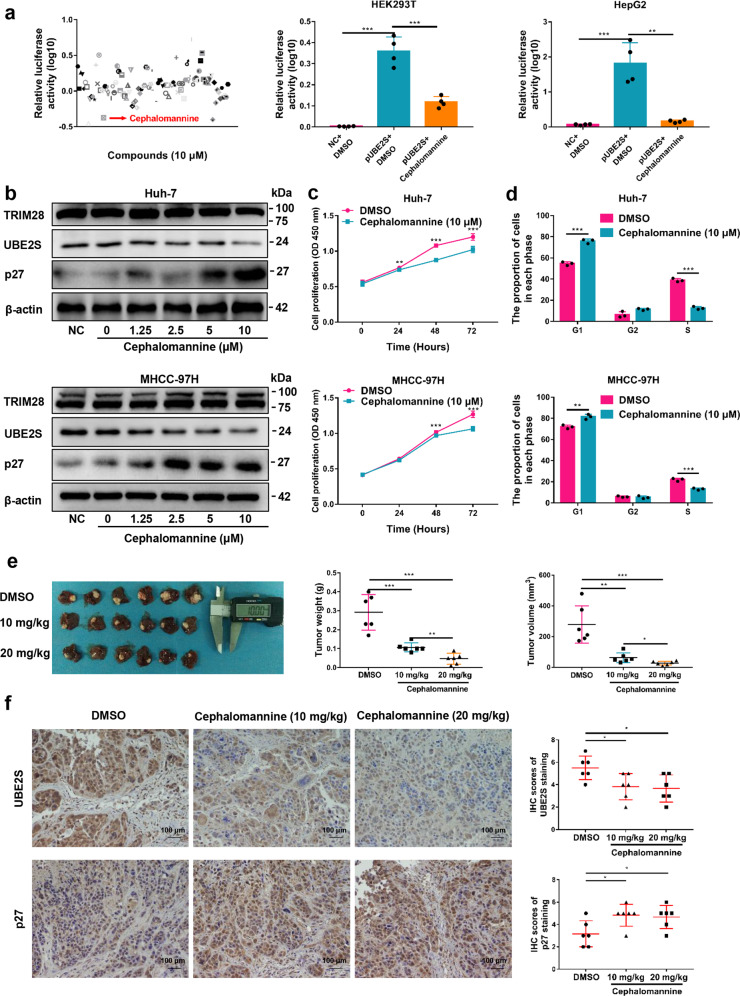
Cephalomannine inhibits UBE2S expression and attenuates HCC growth in vitro and in vivo. **a** Promoter activity of UBE2S in the presence of 100 compounds following 24 h treatment (left) assessed by luciferase reporter assays in HEK293T cells transfected with the pGV238 vector containing human UBE2S promoter (−2000/−1) plasmids. The promoter activity of UBE2S in the presence of cephalomannine (10 μM) for 24 h (middle and right) was detected by luciferase reporter assays in HEK293T and HepG2 cells. **b** Expression of TRIM28, UBE2S, and p27 in HCC cells in the presence of varying concentrations of cephalomannine for 48 h detected by western blot analysis. **c–d** Effects of cephalomannine on cell proliferation and cell cycle progression in HCC cells determined by CCK-8 and flow cytometry assays. **e** Orthotropic tumor-bearing mouse livers and tumor weights and volume in each group (*n* = 6). **f** Expression of UBE2S and p27 in tumor tissues detected by immunohistochemistry analysis. Two-tailed Student’s *t* tests were used to test the significance of differences between two groups; data are represented as mean ± SEM (**a**, **c–f**). *P < 0.05, **P < 0.01, ***P < 0.001

To identify inhibitors of UBE2S that can attenuate tumor progression, we developed a luciferase-based high-throughput screen of small molecule compounds. Luciferase reporter assays showed that cephalomannine significantly inhibited UBE2S promoter activity among the 100 compounds assessed (Fig. 5a). These results were further verified in HEK293T and HepG2 cells (Fig. 5a). In addition, the expression of UBE2S was inhibited by cephalomannine in a concentration-dependent manner in all three of the HCC cell lines assessed (Fig. 5b; Supplementary Fig. 10a).

To explore the effects of cephalomannine on HCC growth, cell proliferation and cell cycle analysis were performed. We found that cephalomannine significantly inhibited tumor cell growth and arrested G1/S progression in HCC cells (Fig. 5c, d; Supplementary Fig. 10b, c). Next, orthotropic transplantation tumor models of MHCC-97H cells in mice were constructed to confirm the role of cephalomannine in vivo. The results showed that the tumor weight and volume were significantly reduced by cephalomannine treatment with dose-dependence compared with control groups, whereas the body weight of tumor-bearing nude mice had no significant difference (Fig. 5e; Supplementary Fig. 11). In addition, immunohistochemical assays showed that the expression of UBE2S in the tumor tissues of the treatment group was significantly lower than that of the control group, whereas the expression of p27 was significantly increased (Fig. 5f). Taken together, these data highlight that cephalomannine can significantly attenuate HCC growth by inhibiting the expression of UBE2S both in vitro and in vivo in a dose-dependent manner.

## Discussion

Hepatocarcinogenesis is a process of sequential selection and change that depends on the accumulation of multiple genes and epigenomic alterations. The researchers have mapped genetic landscape of HCC by high-throughput sequencing technology and have identified ~50–70 protein-altering mutations per tumor.^33^ Most of them are passenger mutations that accumulate randomly and are not involved in carcinogenesis, and only a small subset of mutations are considered to be driver mutations involved in the malignant transformation of tumors.^1^ At present, driver mutated genes and mutation frequency commonly found in HCC are: TERT (50–60%), CTNNB1 (30%), TP53 (30–40%), CDKN2A (10%), CCND1 (10–15%), FGF19 (10–15%), ARID2 (10–15%), etc., and these genes are mainly involved in telomere maintenance, Wnt/β-catenin pathway, TP53/cell cycle pathway, Ras/ERK pathway, and chromatin regulators.^2^ In this study, we identified two novel somatic mutations of UBE2S (p.Gly57Ala and p.Lys63Asn) by WES for the first time, and these two mutations have not been reported in the COSMIC and ICGC databases, thus enriching the current UBE2S mutation landscape in HCC. Although we have not conducted in-depth research on the molecular mechanism of mutant UBE2S, we found these two amino-acid substitutions were potentially deleterious by predictions of the functional effects using scale-invariant feature transform. These results suggest that the UBE2S mutations we identified may be causally implicated in HCC and are worthy of further exploration. Until recently, only a few studies have reported that wild-type UBE2S is highly expressed in HCC and positively correlated with the poor prognosis of patients.^19,34^ As the function and mechanism of UBE2S in HCC are not yet clear, we have focused on the research of wild-type UBE2S rather than mutant-type. Interestingly, we discovered that nuclear UBE2S content was also significantly higher in HCC compared with adjacent tissues and closely correlated with the clinical outcome of HCC patients. These results also lead us to further study the molecular mechanism of nuclear UBE2S promoting HCC progression.

The nucleus of eukaryotic cells is protected by a nuclear membrane, in which the nuclear pore complex (NPC) is the major channel for nucleocytoplasmic transport. Depending on the size of the molecules, NLS-dependent active transport and free diffusion are the primary means through which molecules pass through the NPC.^35^ Although molecules of up to ~50 kDa may pass through NPC by diffusion, small proteins may still require NLS for efficient targeting.^36^ UBE2S is a ~24 kDa protein, and how it passes through NPC is unclear. In this study, we identified a potential NLS sequence at amino-acid position 97–107 of UBE2S using cNLS Mapper. Although UBE2S still has the possibility to enter the nucleus through free diffusion, we believe that the NLS sequence of UBE2S also has a certain effect on its nuclear entry. Moreover, after the key amino acids of NLS were destroyed, UBE2S nuclear entry was reduced, resulting in reduced cell proliferation and G1/S transition. These results reveal new insights into the mechanisms of nuclear UBE2S translocation and its role during HCC progression.

Cell cycle deregulation leads to excessive cell proliferation and malignancy.^37,38^ Previous studies demonstrated that UBE2S could assemble Lys11-linked ubiquitin chains on substrates of the APC/C to enhance spindle formation and mitosis.^39^ In this study, we focused on how UBE2S regulates cell cycle transition in the nucleus. Mass spectrometry of UBE2S-containing complexes in the nucleus indicated that a large number of proteins, such as ILF3, IGF2BP1, ANAX2, and TRIM28, are associated with UBE2S. In particular, we chose TRIM28 that ranked the top one of E3s family in the listed proteins. We found that UBE2S and TRIM28 negatively correlated with p27 expression, and that the levels of p27 ubiquitination significantly increased when UBE2S or TRIM28 were overexpressed. Importantly, we found that UBE2S, TRIM28, and p27 could interact with each other in different HCC cells. Considering the cascading mode of ubiquitination, these results indicate that UBE2S and TRIM28 may act as E2s and E3s, respectively, to enhance the ubiquitination of p27, thus promoting cell cycle progression in HCC. Rupali et al.^40^ reported that the degradation of protein occurs not in the cytosol but in the nucleus. Although we have found UBE2S could interact with TRIM28 directly in the nucleus by the FRET experiment, the place where p27 is eventually degraded after being modified by ubiquitination is still unknown, and it is worth exploring further.

HCC is characterized by poor prognosis and high mortality. Compared with conventional strategies, small-molecule inhibitors are more acceptable in the treatment of tumors including HCC, owing to their low cost, specificity and ease of administration.^41,42^ By screening a variety of compounds, we discovered that cephalomannine could significantly inhibit the expression of UBE2S and attenuate cell growth in various HCC lines and tumor growth in vivo. However, whether cephalomannine only specifically inhibits UBE2S expression is unclear and its potential off-target effects, optimal concentrations, half-life and toxic side effects require further assessment in future studies.

**Fig. 6:**
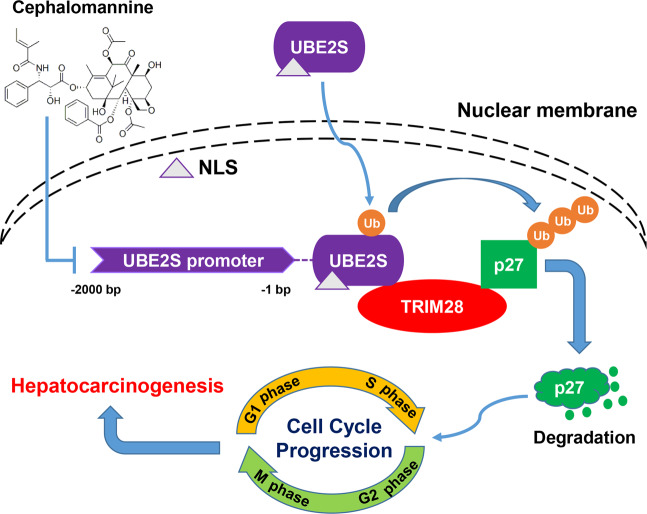
Schematic of the molecular network

In summary, our findings enriched the current UBE2S mutation landscape and revealed the oncogenic activity of wide-type UBE2S in HCC. We believed that UBE2S, especially in the nucleus, might hold value as a prognostic indicator in patients with HCC. In particular, UBE2S could interact with TRIM28 to facilitate the ubiquitination of p27, thus promoting cell cycle progression. Moreover, we identified a small molecule inhibitor cephalomannine, which could inhibit UBE2S expression, providing a new strategy for HCC therapy (Fig. 6).

## Materials and methods

### Patients and samples

Six pairs of punctured HCC and adjacent tissues for WES were from the Youan Hospital, Capital Medical University of China. The samples used in the investigations were obtained from patients with HBV infection who did not receive radiotherapy or chemotherapy prior to radio frequency ablation. In addition, the 80 pairs of HCC and adjacent tissues were obtained from the Xijing Hospital of Fourth Military Medical University. Histopathological diagnosis was based on World Health Organization criteria by hematoxylin and eosin staining. The histological grade of tumor differentiation was defined according to the classification proposed by Edmondson and Steiner. All samples were obtained with informed consent. Ethical approval was obtained from the Ethics Committee of the Capital Medical University of China and the Fourth Military Medical University.

### Validation of candidate mutations

Candidate mutations were validated using the Integrated Genomics Viewer (IGV) and were confirmed using Sanger sequencing in paired samples.^43^ The primer sequences of UBE2S for Sanger sequencing were as follows: forward (TGGCACTGTTTGTCTTTCCAG) and reverse (TCACAATCAGACCGTGGAGAC). The website for analyzing the conservation of amino acid residues in different species sequences was https://www.uniprot.org/. The functional effects of the mutations were predicted by scale-invariant feature transform (http://sift.jcvi.org/).

### Cell culture and transfection

Huh-7 cell line was obtained from the Japanese Collection of Research Bioresources (JCRB, Osaka, Japan). HepG2 cell line and HEK293T cell line were obtained from the American Type Culture Collection (Manassas, VA, USA). MHCC-97H cell line was obtained from the Xijing Hospital of Digestive Diseases of the Fourth Military Medical University. All cell lines were cultured in RPMI 1640 medium supplemented with 10% fetal bovine serum, 1% penicillin, and streptomycin in 5% CO_2_ at 37 °C. Cells were transfected with plasmids or siRNA using Lipofectamine 2000 (Invitrogen) according to the manufacturer’s instructions. Cells were transiently transfected for 24 h or 48 h for mRNA and protein assessments, respectively. Sequences for siRNAs were listed in Supplementary Table 2.

### Cell proliferation assays

Huh-7, HepG2, and MHCC-97H cells with UBE2S silencing or overexpression were seeded into 96-well plates (100 µl cell suspensions). Cell numbers were assessed every 24 h by CCK-8 assays according to the manufacturer’s instructions.

### Cell invasion and migration assays

Cell invasion and migration assays were performed in 24-well plates with polyethylene terephalate membrane filters separating the lower and upper culture chambers. Matrigel was added to the upper chambers for cell invasion assays. In brief, 0.5 × 10^5^ (MHCC-97H) or 1 × 10^5^ (Huh-7 or HepG2) cells were added to the upper chambers in serum-free RPMI 1640 medium, and 10% FBS was added to the lower chambers. After 16–24 h (MHCC-97H) or 36–48 h (Huh-7 or HepG2), chambers were fixed for 15 min and stained with 0.1% crystal violet for 20 min and imaged.

### Immunohistochemistry and immunofluorescence

For immunohistochemistry, tissues were fixed in 10% formalin and paraffin embedded. Sections were deparaffinized in xylene and alcohol, and antigen retrieval was performed using citrate buffer (pH 6.0). Sections were blocked in H_2_O_2_ and normal goat serum and probed with primary antibodies at 4 °C overnight in a moist chamber. Sections were then stained using a streptavidin-peroxidase staining kit (Zhongshan Jinqiao Co., Beijing, China). In human HCC and adjacent tissues, if the proportion of positive staining of nuclear UBE2S in observed field is <10%, the tissue is considered nuclear UBE2S negative. For immunofluorescence assay, cells in dishes were fixed in 4% formaldehyde and probed with primary antibodies overnight at 4 °C. After incubation, cells were stained for 1.5 h with Alexa 555-conjugated donkey anti-mouse IgG. Cell nuclei were stained with 4’,6-diamidino-2-phenylindole. Cells were imaged on a confocal microscope using Nikon NIS-Elements software (Nikon, Tokyo, Japan). Primary antibodies were listed in Supplementary Table 3.

### Luciferase reporter assay

The UBE2S promoter vector (pGV238-UBE2S) was purchased from Genechem Co., Ltd. and contained the firefly luciferase reporter and UBE2S promoter (−2000/−1). The pGV238-UBE2S vector was co-transfected with the internal control pRL-TK (Promega, Madison, WI, USA) in HEK293T cells or HepG2 cells using Lipofectamine 2000 (Invitrogen). Twenty-four hours post transfection, cells in the presence or absence of small molecule compounds were assessed for luciferase activity using the Luciferase Reporter Assay System (Promega) according to the manufacturer’s protocol. Small molecule compounds were listed in Supplementary Table 4.

### Orthotropic xenograft models

For xenograft models, 3 × 10^6^ cells were subcutaneously inoculated into nude mice. After 3 weeks, nude mice were euthanized and same size tumor mass was inoculated into the liver capsules of other mice. Two-weeks later, different concentrations of cephalomannine and dimethyl sulfoxide were injected intraperitoneally into the mice (four injections in total, once every 3 days).

### Statistical analysis

Data were obtained from three independent experiments and are expressed as the mean ± SEM. Data were analyzed using GraphPad Prism v7.0 software (GraphPad Software, La Jolla, CA, USA) and SPSS 23.0 (SPSS, Inc., Chicago, IL, USA). OS rate was calculated using Kaplan–Meier analysis. The correlation between the UBE2S expression and clinicopathological variables was computed using *χ*^2^ tests. Mean values were compared by a Student’s *t* test. The correlation between UBE2S and TRIM28 in HCC tissue was performed through Pearson’s correlation analysis. *P* < 0.05 was considered statistically significant.

## Supplementary information

Supplementary File

## Data Availability

The data sets used and/or analyzed during the current study are available from the corresponding author on reasonable request.
