# Supplementary material for: UBE2S interacting with TRIM28 in the nucleus accelerates cell cycle by ubiquitination of p27 to promote hepatocellular carcinoma development
Source: Signal Transduct Target Ther. 2021 Feb 16;6:64. doi: 10.1038/s41392-020-00432-z (PMC7884418; doi:10.1038/s41392-020-00432-z)
Supplement: Supplementary file 1 — Supplementary File [file 41392_2020_432_MOESM1_ESM.docx]

Supplementary Materials for

UBE2S interacting with TRIM28 in the nucleus accelerates cell cycle by ubiquitination of p27 to promote hepatocellular carcinoma development

Ren-Yu Zhang^1#^, Ze-Kun Liu^1#^, Ding Wei^1^, Yu-Le Yong^1^, Peng Lin^1^, Hao Li^1^, Man Liu^1^, Nai-Shan Zheng^1^, Ke Liu^2^, Cai-Xia Hu^3^, Xiao-Zhen Yang^3^, Zhi-Nan Chen^1^*, Huijie Bian^1^*

Correspondence to: hjbian@fmmu.edu.cn; huijiebian@hotmail.com

znchen@fmmu.edu.cn

**This file includes:**

Figures S1 to S11

Tables S1 to S4

**Figure S1.**

**
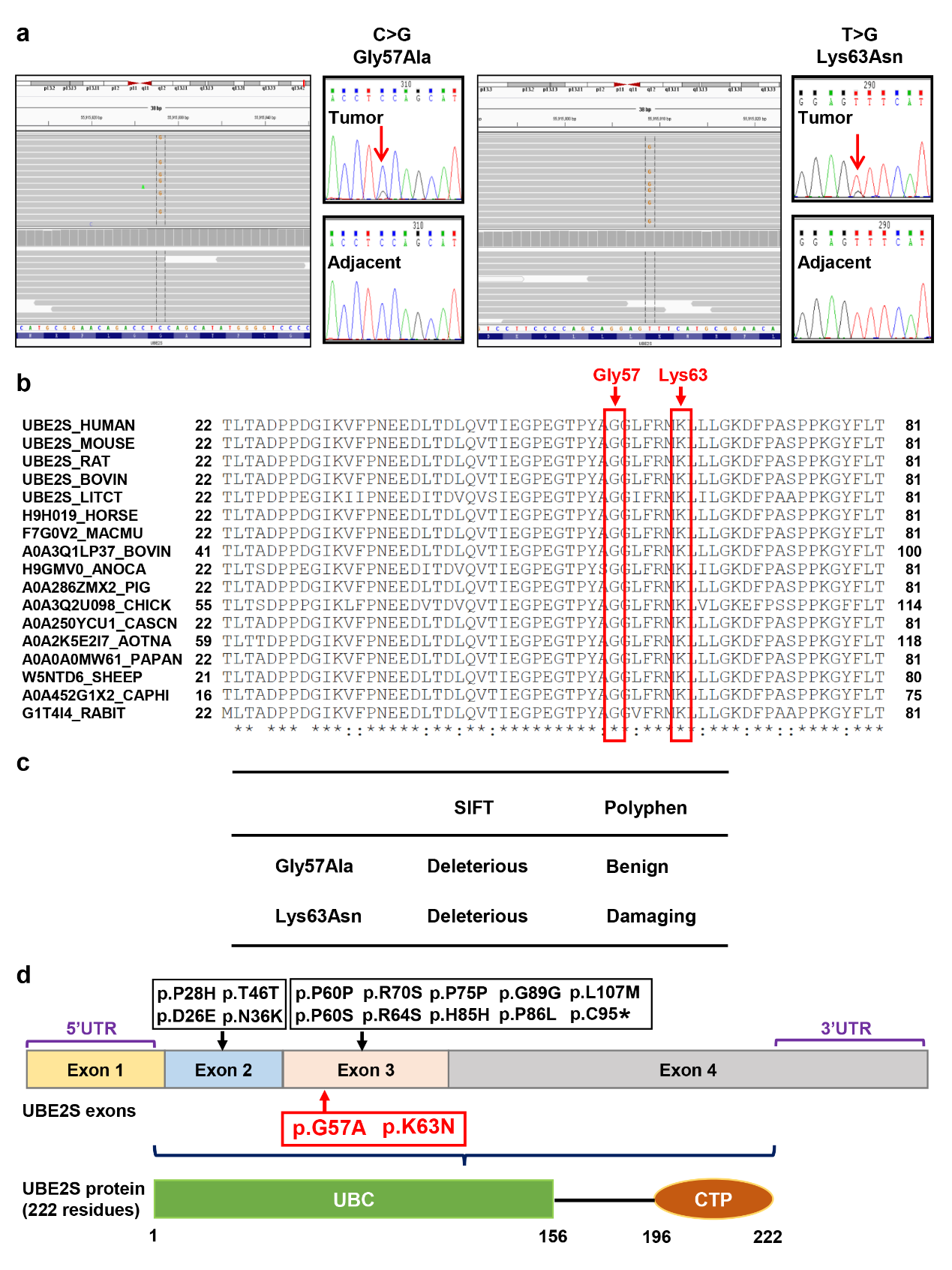
**

**Supplementary Fig. 1** UBES2 mutations in HCC. (**a**) Somatic mutations identified in UBE2S were confirmed by IGV visualization and Sanger sequencing of DNA samples from HCC and adjacent non-tumor tissue. Red arrows indicate the somatic mutation sites. (**b**) Multiple sequence alignments of UBE2S paralogs from different species. (**c**) Prediction of the functional effects of Gly57Ala and Lys63Asn substitutions. (**d**) Genomic organization of UBE2S exons and protein domains. Black arrows indicate the somatic mutation sites reported in the COSMIC database and ICGC database. Red arrows indicate the somatic mutation sites identified in this study.

**Figure S2.**


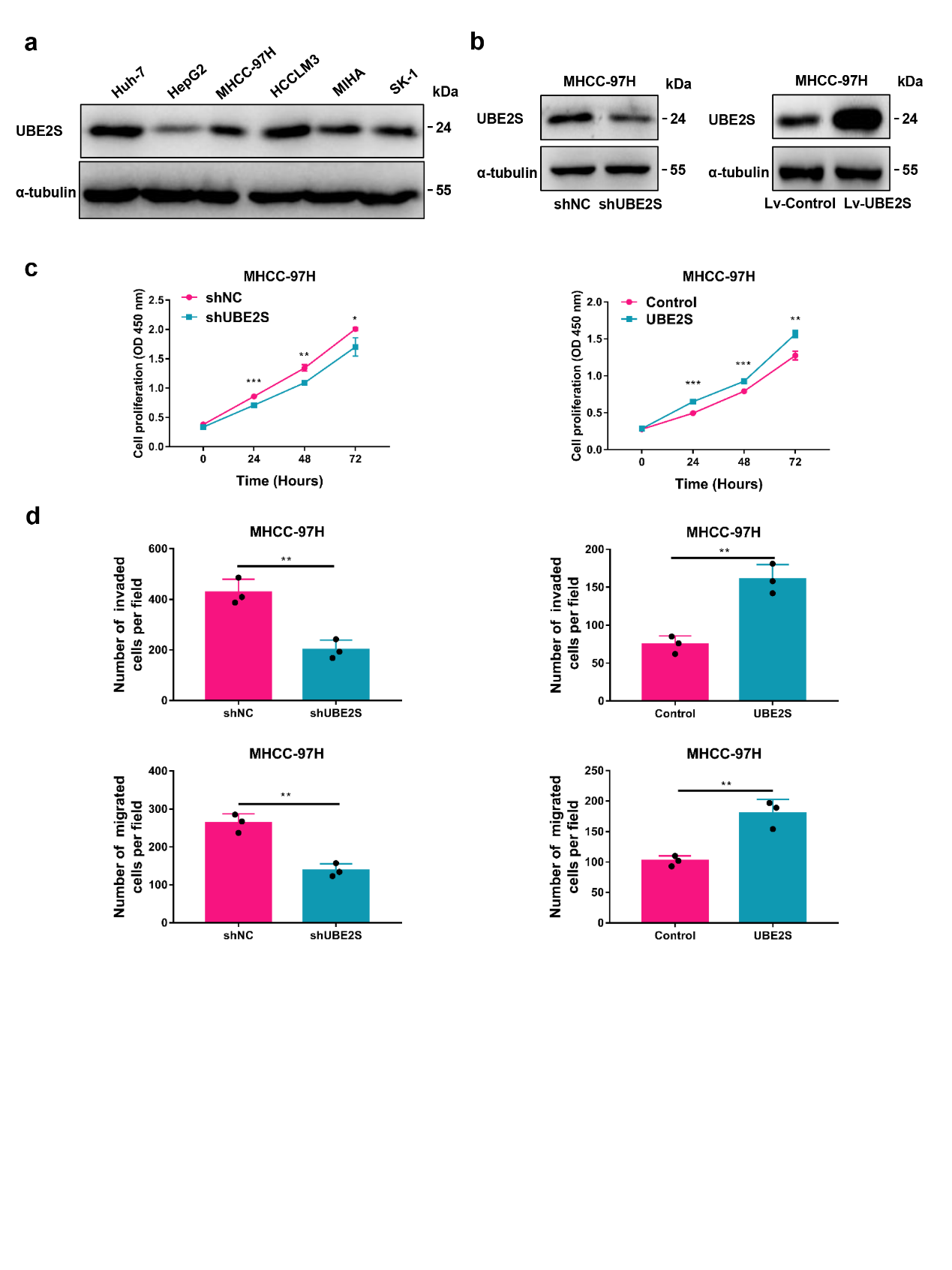
**Supplementary Fig. 2** UBE2S promotes proliferation, invasion and migration in MHCC-97H cells. **(a)** Expression of UBE2S in different HCC and normal hepatic cells detected by western blot analysis. **(b)** Expression of UBE2S in MHCC-97H cells infected with shUBE2S or UBE2S lentiviruses detected by western blot analysis. **(c)** Effects of UBE2S on cell proliferation determined by CCK-8 assays. **(d)** Effects of UBE2S on cell invasion and migration evaluated by transwell assays. Two-tailed Student’s t-tests were used to test the significance of differences between two groups; data are represented as mean ± SEM (**c–d**). **P* < 0.05, ***P* < 0.01, ****P* < 0.001.

**Figure S3.**

**
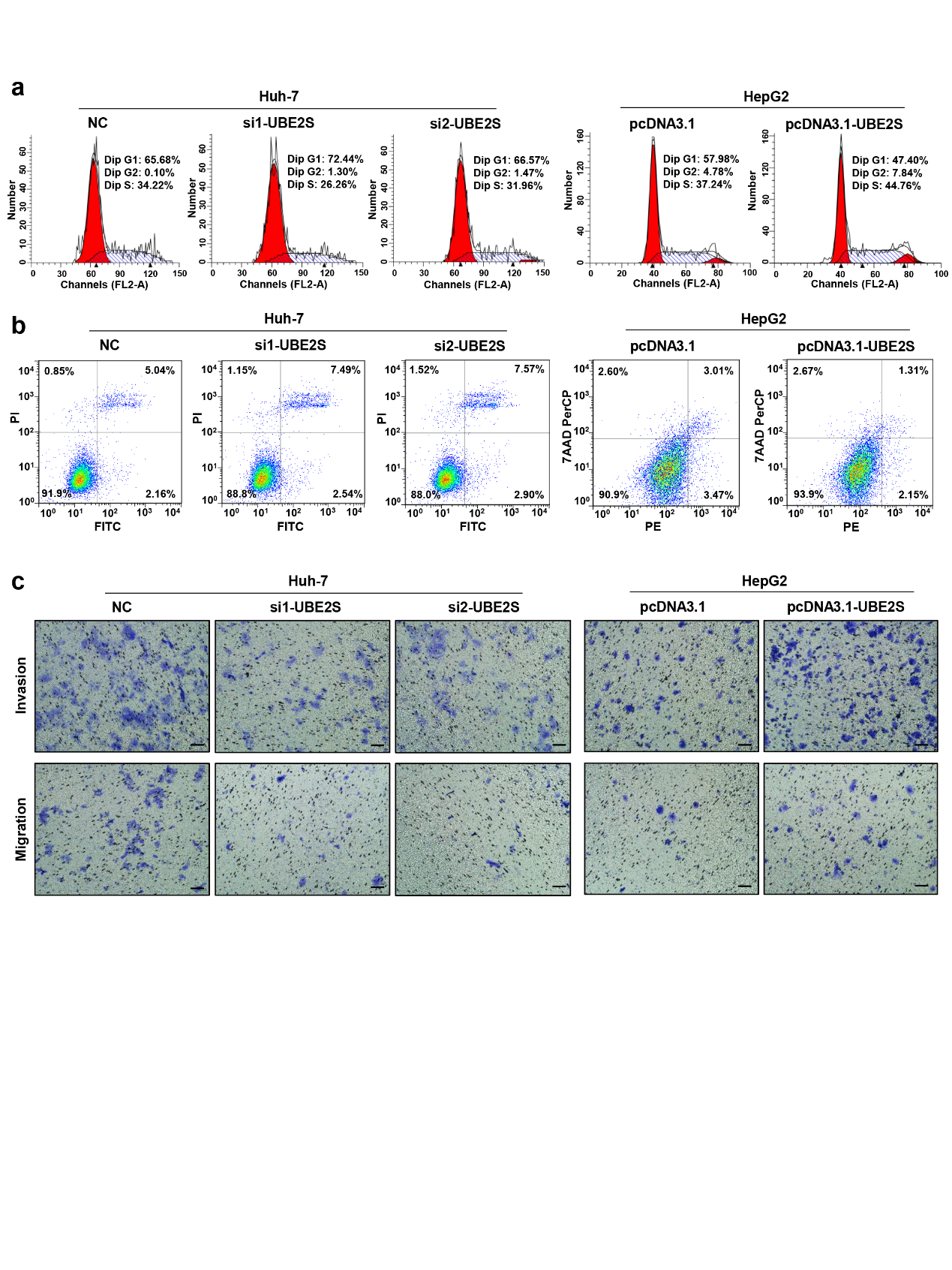
**

**Supplementary Fig. 3** The flow cytometry analysis of cell cycle distribution (**a**) and cell apoptosis (**b**) and transwell (**c**). Scale bars: 100 μm.

**Figure S4.**

**
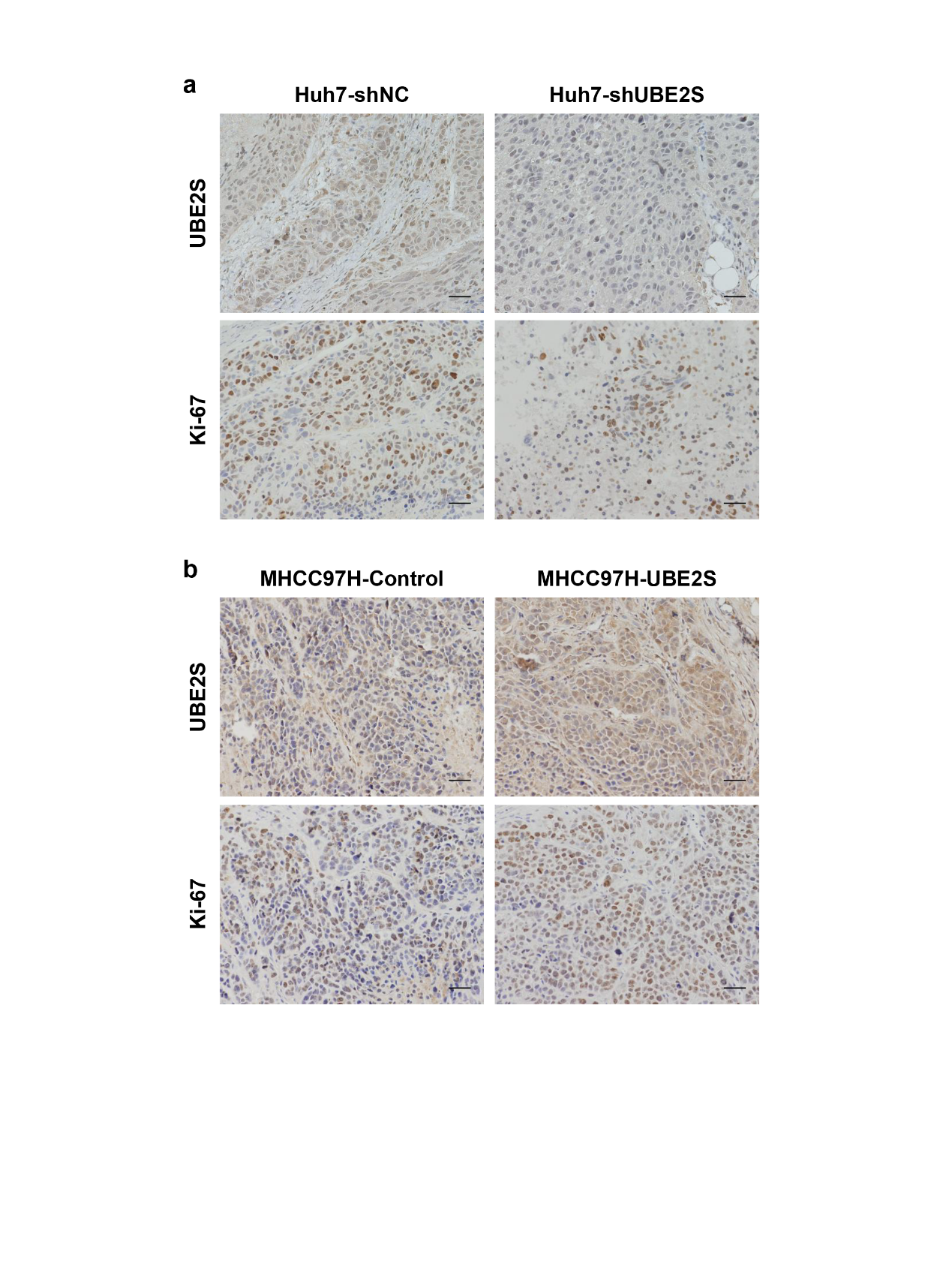
**

**Supplementary Fig. 4** Immunohistochemical detection of UBE2S and Ki-67 expression in tumor tissues of xenograft nude mouse models with UBE2S knockdown (**a**) or overexpression (**b**). Scale bars: 100 μm.

**Figure S5.**

**
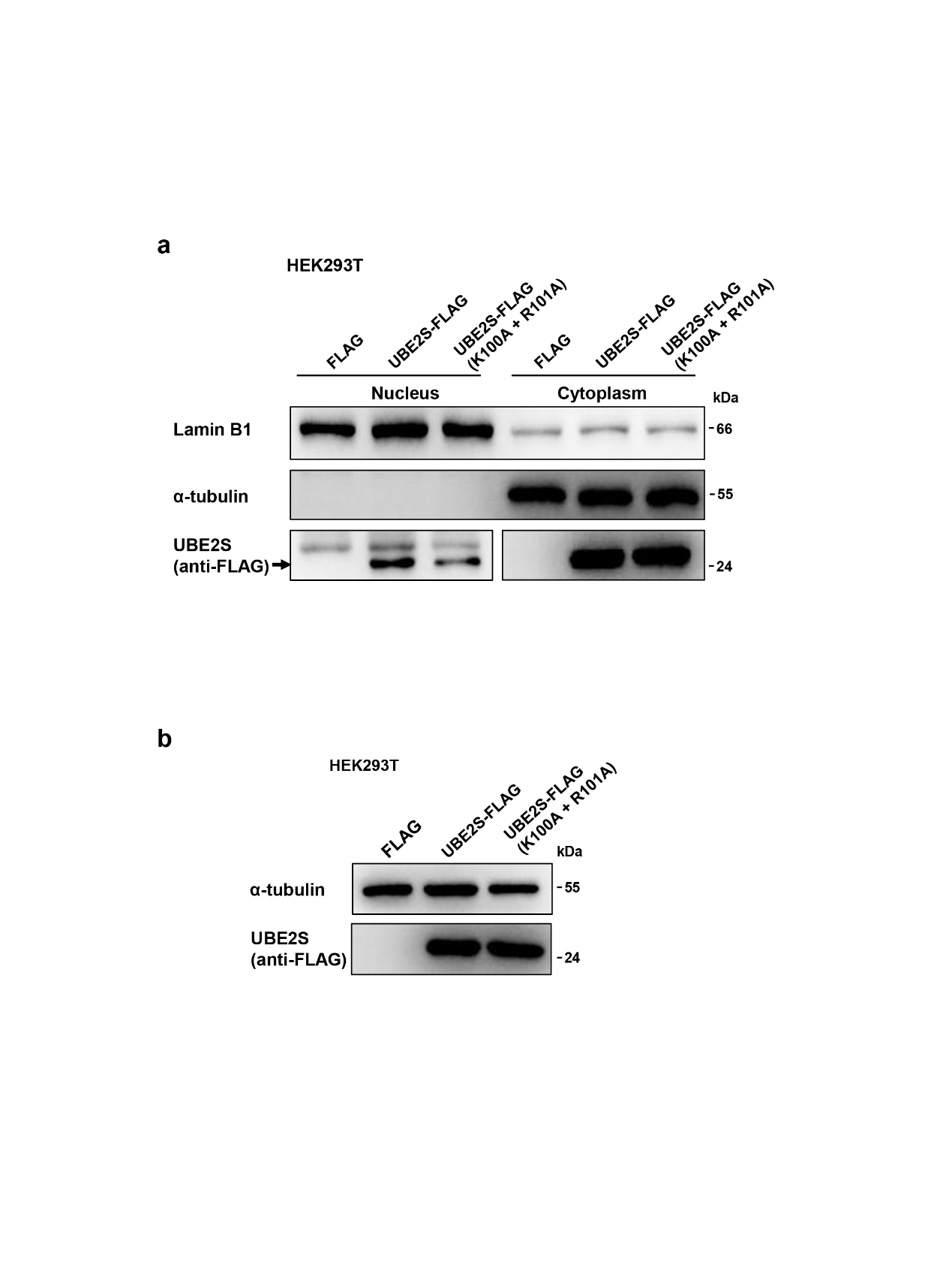
**

**Supplementary Fig. 5** UBE2S-FLAG expression in the nuclear or cytoplasmic fractions of cell lysates (**a**) and total lysates (**b**) in HEK293T cells transiently transfected with UBE2S-FLAG NLS mutation plasmids assessed by western blotting.

**Figure S6.**

**
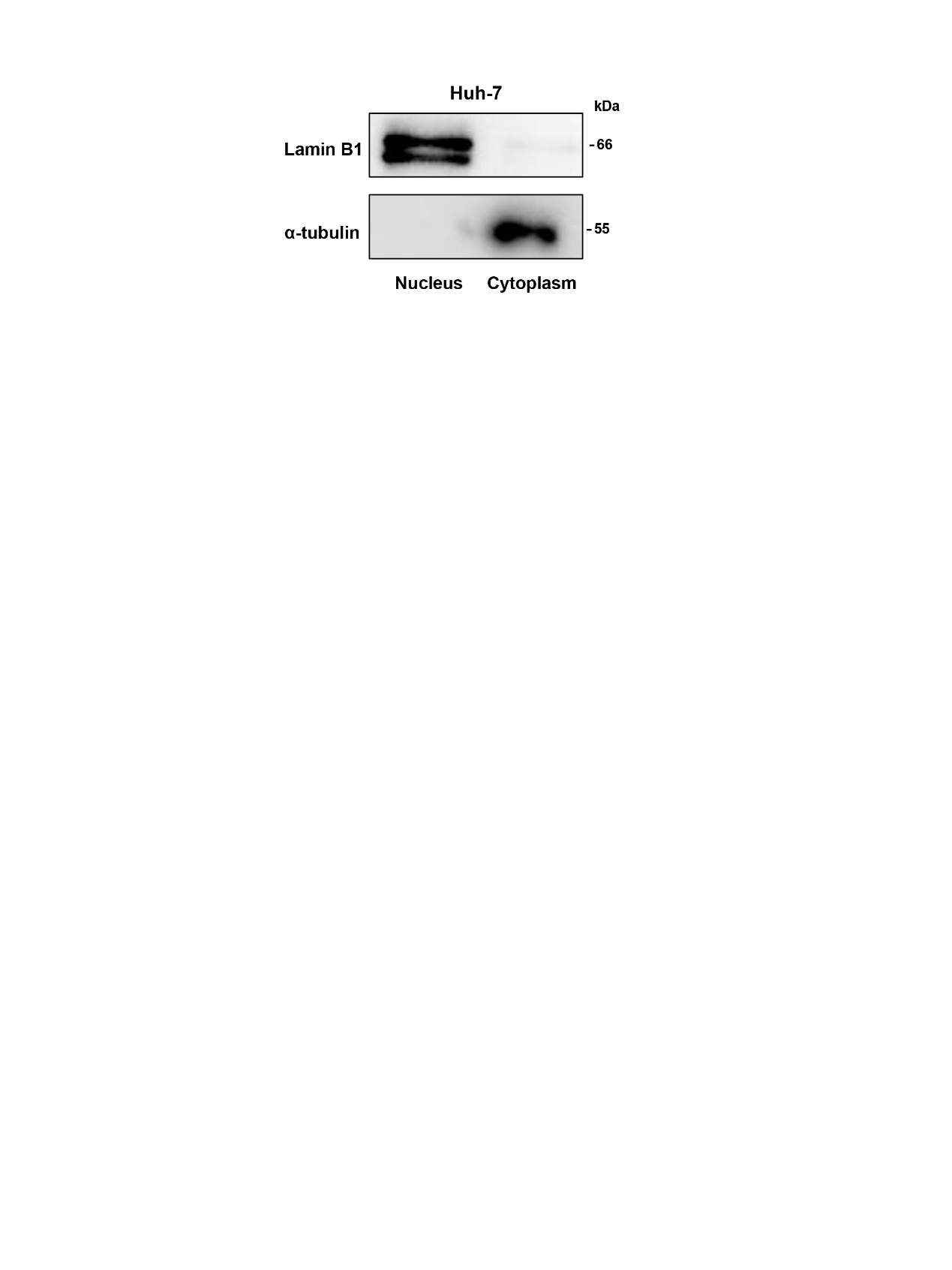
**

**Supplementary Fig. 6** Nucleus/cytoplasmic fractionations in Huh-7 cells. Western blotting analysis was used to detect the expression of Lamin B1 and α-tubulin in the nucleus and cytoplasm of Huh-7 cells, respectively.

**Figure S7.**


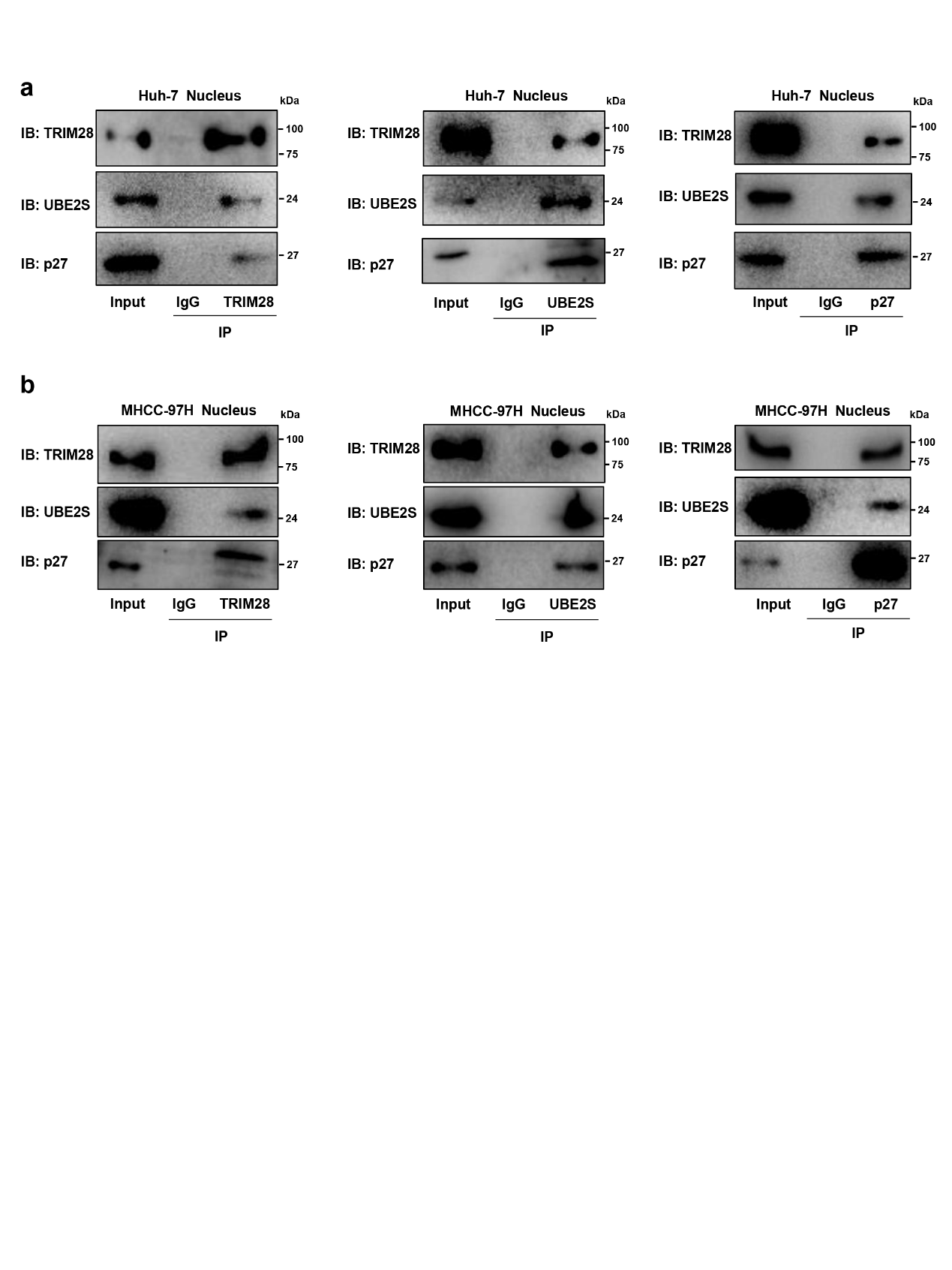


**Supplementary Fig. 7** Endogenous interaction of UBE2S, TRIM28 and p27 in nuclear extract of Huh-7 cells (**a**) and MHCC-97H cells (**b**).

**Figure S8.**

**
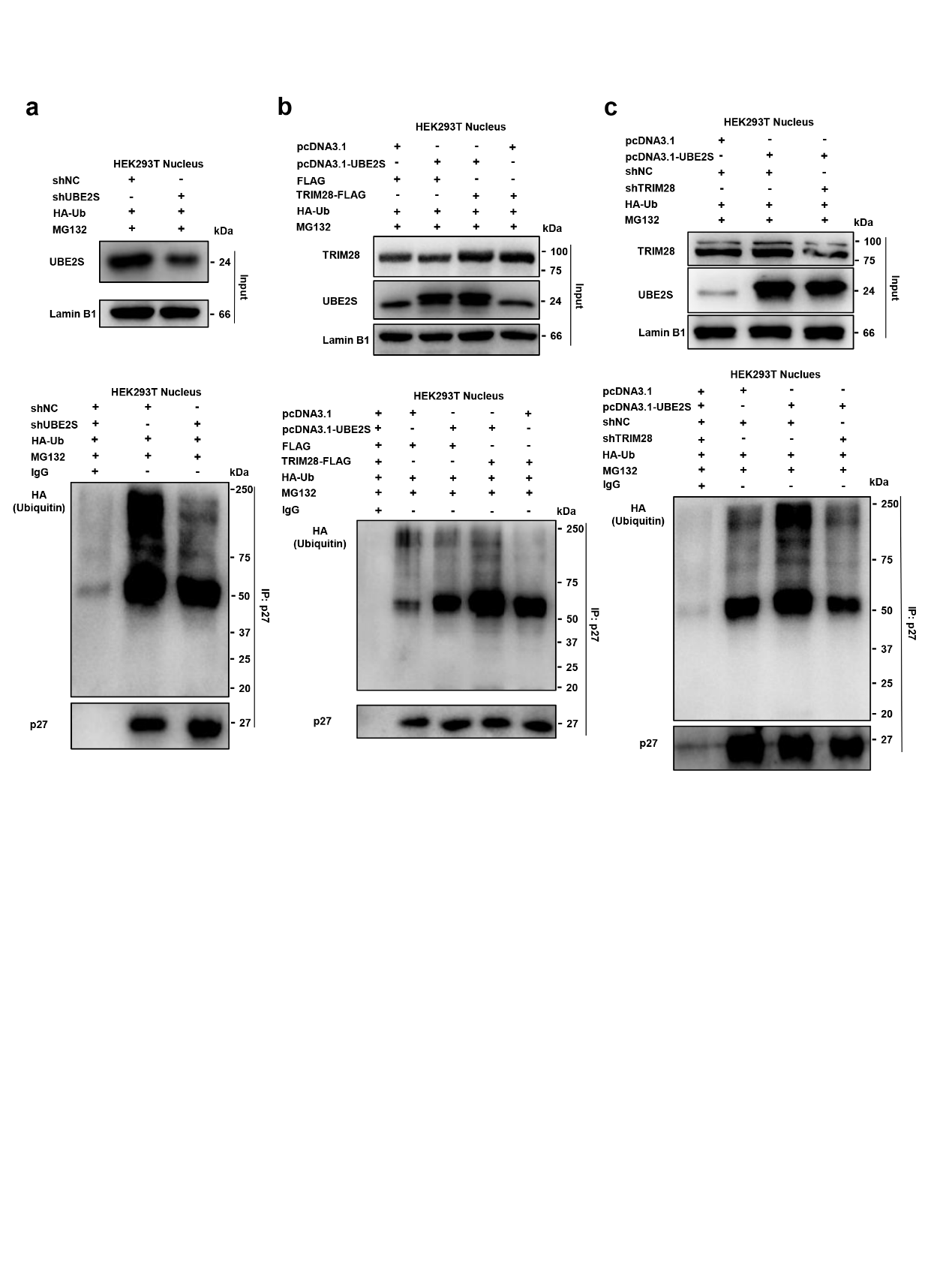
**

**Supplementary Fig. 8** UBE2S and TRIM28 enhance the ubiquitination of p27 in the nucleus. Ubiquitination of p27 in HEK293T cells with UBE2S and TRIM28 overexpression or knockdown in the presence of MG132 (10 μM, 12 h) detected by immunoprecipitation and western blot analysis.

**Figure S9.**


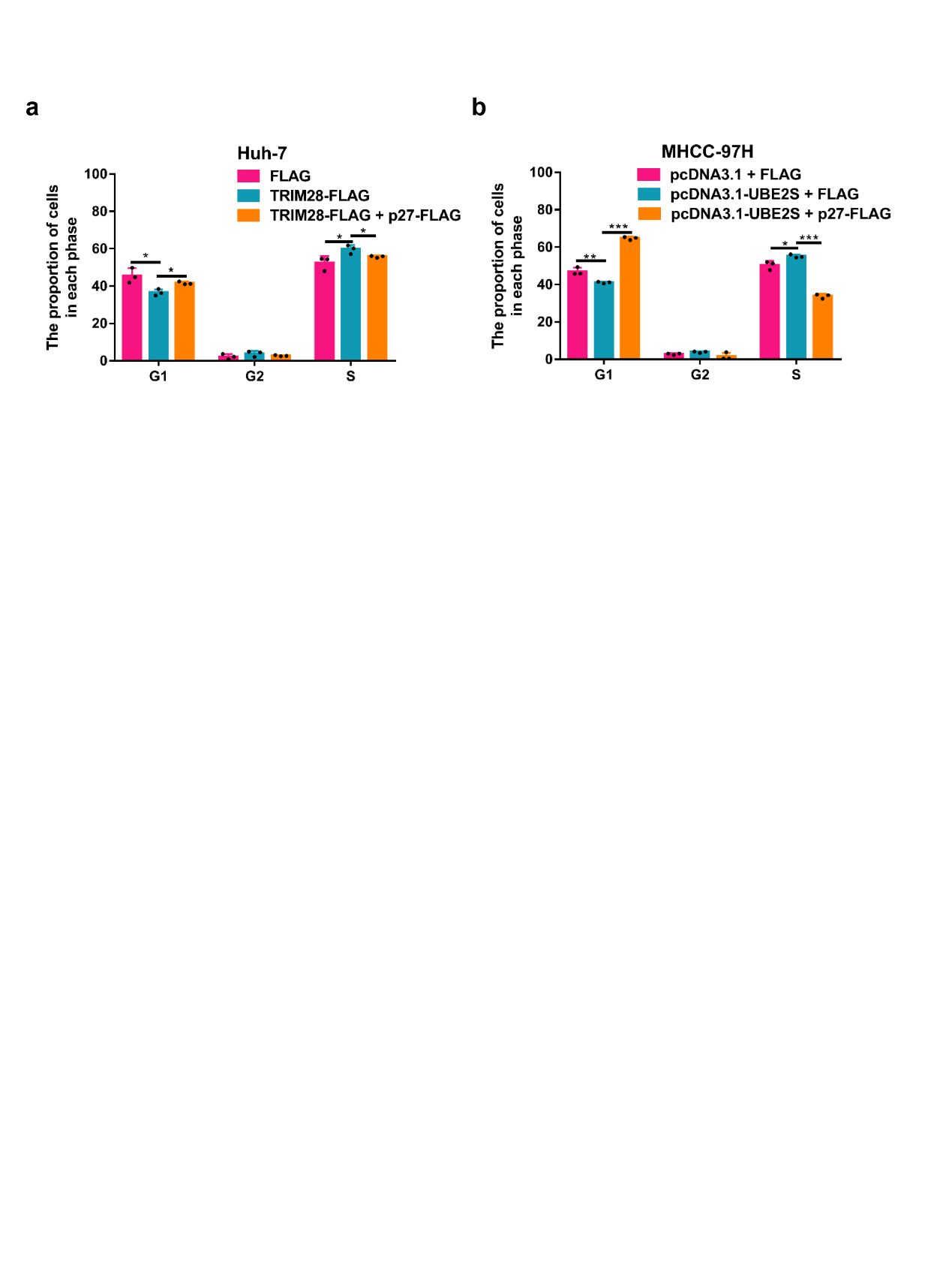


**Supplementary Fig. 9** Effects of UBE2S/TRIM28 and p27 on cell cycle progression detected by flow cytometry assays in Huh-7 cells (**a**) and MHCC-97H cells (**b**). Two-tailed Student’s t-tests were used to test the significance of differences between two groups; data are represented as mean ± SEM (**a–b**). **P* < 0.05, ***P* < 0.01, ****P* < 0.001.

**Figure S10.**


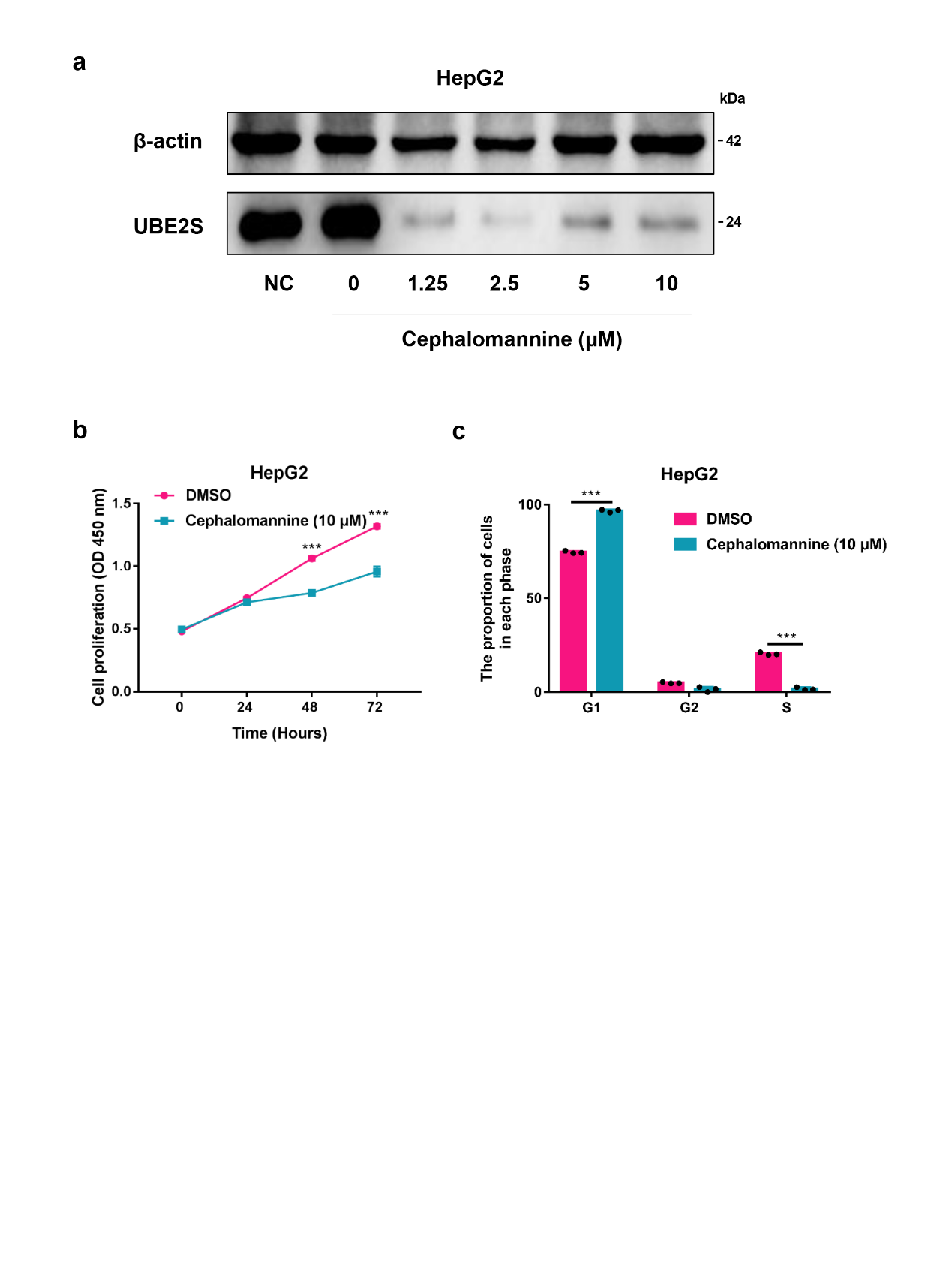


**Supplementary Fig. 10** Cephalomannine inhibits UBE2S expression and attenuates cell growth in HepG2 cells. **(a)** Expression of UBE2S in HepG2 cells in the presence of different concentrations of cephalomannine for 48 h detected by western blot analysis. **(b, c)** Effects of cephalomannine on cell proliferation and cell cycle progression in HepG2 cells determined by CCK-8 and flow cytometry assays. Two-tailed Student’s t-tests were used to test the significance of differences between two groups; data are represented as mean ± SEM (**b–c**). ****P* < 0.001.

**Figure S11.**


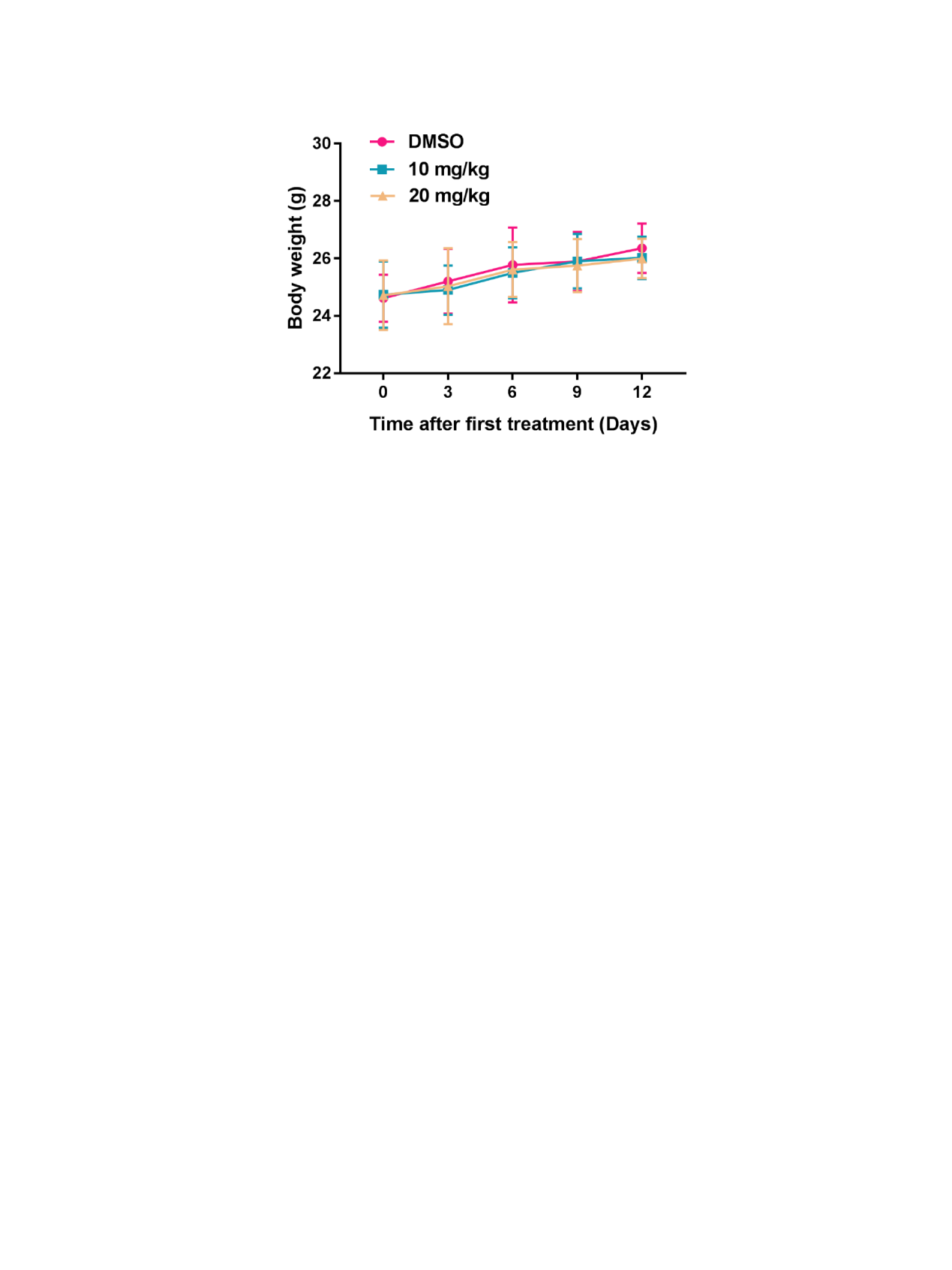


**Supplementary Fig. 11** The body weight of tumor-bearing nude mice during cephalomannie treatment. Two-tailed Student’s t-tests were used to test the significance of differences between two groups; data are represented as mean ± SEM.

**Table S1.**

**Supplementary Table 1 The list of UBE2S binding proteins identified by IP-MS**

| No. | Name | Coverage | PSMs | Peptides | MW [kDa] | Score |
| --- | --- | --- | --- | --- | --- | --- |
| 1 | Interleukin enhancer-binding factor 3 (ILF3) | 31.1 | 39 | 18 | 95.3 | 200.24 |
|  |  |  |  |  |  |  |
| 2 | Stress-70 protein, mitochondrial (HSPA9) | 34.17 | 47 | 16 | 73.6 | 195.33 |
| 3 | 78 kDa glucose-regulated protein (HSPA5) | 31.04 | 40 | 15 | 72.3 | 159.08 |
| 4 | Glutamate dehydrogenase 1, mitochondrial (GLUD1) | 26.88 | 34 | 12 | 61.4 | 151.64 |
| 5 | Isoform 2 of heat shock cognate 71 kDa protein (HSPA8) | 31.03 | 31 | 11 | 53.5 | 130.33 |
| 6 | Insulin-like growth factor 2 mRNA-binding protein 1 (IGF2BP1) | 28.42 | 25 | 11 | 63.4 | 119.14 |
| 7 | Annexin A2 (ANXA2) | 38.64 | 40 | 10 | 38.6 | 201.32 |
| 8 | Isoform 3 of peroxisomal multifunctional enzyme type 2 (HSD17B4) | 23.4 | 10 | 10 | 77.8 | 54.93 |
| 9 | Nucleolin (NCL) | 16.2 | 32 | 9 | 76.6 | 146.16 |
| 10 | Isoform short of heterogeneous nuclear ribonucleoprotein U (HNRNPU) | 19.73 | 30 | 9 | 88.9 | 141.97 |
| 11 | 3-hydroxyacyl-CoA dehydrogenase type-2 (HSD17B10) | 56.32 | 20 | 9 | 26.9 | 85.52 |
| 12 | Isoform C1 of heterogeneous nuclear ribonucleoproteins C1/C2 (HNRNPC) | 40.27 | 34 | 8 | 32.3 | 147.87 |
| 13 | Actin, cytoplasmic 1 (ACTB) | 40.53 | 23 | 8 | 41.7 | 134.86 |
| 14 | Isoform 2 of transcription intermediary factor 1-beta (TRIM28) | 20.85 | 19 | 8 | 79.4 | 105.2 |
| 15 | Isoform 2 of glyceraldehyde-3- phosphate dehydrogenase (GAPDH) | 55.97 | 20 | 8 | 31.5 | 92.53 |
| 16 | Interleukin enhancer-binding factor 2 (ILF2) | 31.28 | 23 | 8 | 43 | 91.56 |
| 17 | Isoform 3 of serine hydroxymethyltransferase, mitochondrial (SHMT2) | 24.43 | 11 | 8 | 53.4 | 76.94 |
| 18 | RNA-binding motif protein, X chromosome (RBMX) | 22.51 | 20 | 7 | 42.3 | 119.91 |
| 19 | Prelamin-A/C (LMNA) | 17.47 | 14 | 7 | 74.1 | 63.49 |
| 20 | Isoform 6 of SWI/SNF-related matrix-associated actin-dependent regulator of chromatin subfamily E member 1 (SMARCE1) | 36.86 | 15 | 7 | 34.4 | 59.65 |

**Table S2.**

**Supplementary Table 2 The sequences used in this study**

| siRNAs | Sequences |
| --- | --- |
| snc RNA | sense 5’-UUCUCCGAACGUGUCACGUTT-3’ |
|  | antisense 5’-ACGUGACACGUUCGGAGAATT-3’ |
| si1-UBE2S | sense 5’-UCAUCCGCCUGGUGUACAATT-3’ |
|  | antisense 5’-UUGUACACCAGGCGGAUGATT-3’ |
| si2-UBE2S | sense 5’-GACACGUACUGCUGACCAUTT-3’ |
|  | antisense 5’-AUGGUCAGCAGUACGUGUCTT-3’ |
| si1-TRIM28 | sense 5’-GCAACAGUGCUUCUCCAAATT-3’ |
|  | antisense 5’-UUUGGAGAAGCACUGUUGCTT-3’ |
| si2-TRIM28 | sense 5’-GGAGCACAUUCUGCGCUUUTT-3’ |
|  | antisense 5’-AAAGCGCAGAAUGUGCUCCTT-3’ |

**Table S3.**

**Supplementary Table 3 Primary antibodies used for western blotting, immunohistochemistry and immunofluorescence assays**

| Antibody | Company (Cat. No.) | | Molecular  weight (kDa) | Working dilutions |
| --- | --- | --- | --- | --- |
| UBE2S | Proteintech (14115-1-AP) | 24 | | WB: 1/1000 |
| TRIM28 | Proteintech (66630-1-Ig) | 89 | | WB: 1/1000 |
| Lamin B1 | Proteintech (66095-1-Ig) | 66 | | WB: 1/1000 |
| CDK2 | Proteintech (10122-1-AP) | 33 | | WB: 1/1000 |
| CDK4 | Proteintech (11026-1-AP) | 34 | | WB: 1/500 |
| Cyclin D1 | Proteintech (60186-1-Ig) | 34 | | WB: 1/2000 |
| Cyclin E1 | Proteintech (11554-1-AP) | 47 | | WB: 1/500 |
| p27 | Proteintech (25614-1-AP) | 27 | | WB: 1/1000  IHC:1/200 |
| α-tubulin | Proteintech (11224-1-AP) | 55 | | WB: 1/2000 |
| β-actin | Proteintech (66009-1-Ig) | 42 | | WB: 1/2000 |
| HA tag | Proteintech (51064-2-AP) | / | | WB: 1/1000 |
| Flag | Huabio (M1403-2) | / | | WB: 1/1000 |
| Ki67 | Abcam (ab16667) | / | | IHC: 1/200 |
| UBE2S | Abcam (ab197945) | / | | IHC: 1/100 |
| UBE2S | Biorbyt (orb5098) | / | | IF: 1/200 |
| DYKDDDDK Tag | Cell Signaling (8146S) | / | | IF: 1/1000 |

**Table S4.**

**Supplementary Table 4 Product names of 100 small molecule compounds**

| **No.** | **Product Name** | **No.** | **Product Name** |
| --- | --- | --- | --- |
| 1 | Bortezomib | 51 | Cordycepin |
| 2 | Panobinostat | 52 | (+)-Fangchinoline |
| 3 | Vorinostat | 53 | Rosmarinic acid |
| 4 | Entinostat | 54 | Scoparone |
| 5 | Belinostat | 55 | Sesamol |
| 6 | Abexinostat | 56 | Fludrocortisone acetate |
| 7 | Quisinostat 2HCl | 57 | Umbelliferone |
| 8 | Mocetinostat | 58 | Cinnamic acid |
| 9 | Ibutamoren mesylate | 59 | Flavanone |
| 10 | Delanzomib | 60 | Trigonelline Hydrochloride |
| 11 | Thalidomide | 61 | Monocrotaline |
| 12 | Avagacestat | 62 | Tubeimoside I |
| 13 | Costunolide | 63 | Tectoridin |
| 14 | Semagacestat | 64 | Glycitin |
| 15 | Reserpine | 65 | Isoalantolactone |
| 16 | Nitazoxanide | 66 | 6-Gingerol |
| 17 | Tretinoin | 67 | Isoquercitrin |
| 18 | Mitotane | 68 | Eupatilin |
| 19 | Cyclocytidine HCl | 69 | Glucosamine sulfate |
| 20 | Meglumine | 70 | Methyl protocatechuate |
| 21 | Capsaicin | 71 | Hydroxy Camptothecine |
| 22 | Cytidine | 72 | (-)-epigallocatechin |
| 23 | Ixazomib | 73 | Ginsenoside Rd |
| 24 | Ixazomib Citrate | 74 | Bergapten |
| 25 | Dihydroartemisinin | 75 | Etofibrate |
| 26 | Formononetin | 76 | 5,5-Dimethyloxazolidine-2,4-dione |
| 27 | Hesperidin | 77 | Wogonin |
| 28 | Oxymatrine | 78 | Propacetamol hydrochloride |
| 29 | Quercetin Dihydrate | 79 | Propiverine hydrochloride |
| 30 | Sclareol | 80 | Indometacin Sodium |
| 31 | Silibinin | 81 | Indobufen |
| 32 | Silymarin | 82 | Fenretinide |
| 33 | Ursolic Acid | 83 | Canrenone |
| 34 | Dioscin | 84 | Forchlorfenuron |
| 35 | Indirubin | 85 | Dehydroepiandrosterone acetate |
| 36 | Isoliquiritigenin | 86 | D-Ribose |
| 37 | Cephalomannine | 87 | Thioridazine hydrochloride |
| 38 | 10-Deacetylbaccatin-III | 88 | Vitamin A |
| 39 | (S)-10-Hydroxycamptothecin | 89 | Ceftriaxone Sodium |
| 40 | Dimethyl fumarate | 90 | Oprozomib |
| 41 | Clindamycin palmitate HCl | 91 | Idasanutlin |
| 42 | Lonidamine | 92 | Alvelestat |
| 43 | Rabusertib | 93 | CPI-1205 |
| 44 | Nocodazole | 94 | 20S-Ginsenoside Rh2 |
| 45 | Tacedinaline | 95 | Solamargine |
| 46 | Carfilzomib | 96 | Oroxylin A |
| 47 | Daunorubicin HCl | 97 | Schisandrin C |
| 48 | Fosaprepitant dimeglumine salt | 98 | Koumine |
| 49 | Betulinic acid | 99 | Kirenol |
| 50 | Fangchinoline | 100 | Tomatine |
